# Emergence of Functional Hierarchy in a Multiple Timescale Neural Network Model: A Humanoid Robot Experiment

**DOI:** 10.1371/journal.pcbi.1000220

**Published:** 2008-11-07

**Authors:** Yuichi Yamashita, Jun Tani

**Affiliations:** Laboratory for Behavior and Dynamic Cognition, RIKEN Brain Science Institute, Wako-shi, Saitama, Japan; Indiana University, United States of America

## Abstract

It is generally thought that skilled behavior in human beings results from a functional hierarchy of the motor control system, within which reusable motor primitives are flexibly integrated into various sensori-motor sequence patterns. The underlying neural mechanisms governing the way in which continuous sensori-motor flows are segmented into primitives and the way in which series of primitives are integrated into various behavior sequences have, however, not yet been clarified. In earlier studies, this functional hierarchy has been realized through the use of explicit hierarchical structure, with local modules representing motor primitives in the lower level and a higher module representing sequences of primitives switched via additional mechanisms such as gate-selecting. When sequences contain similarities and overlap, however, a conflict arises in such earlier models between generalization and segmentation, induced by this separated modular structure. To address this issue, we propose a different type of neural network model. The current model neither makes use of separate local modules to represent primitives nor introduces explicit hierarchical structure. Rather than forcing architectural hierarchy onto the system, functional hierarchy emerges through a form of self-organization that is based on two distinct types of neurons, each with different time properties (“multiple timescales”). Through the introduction of multiple timescales, continuous sequences of behavior are segmented into reusable primitives, and the primitives, in turn, are flexibly integrated into novel sequences. In experiments, the proposed network model, coordinating the physical body of a humanoid robot through high-dimensional sensori-motor control, also successfully situated itself within a physical environment. Our results suggest that it is not only the spatial connections between neurons but also the timescales of neural activity that act as important mechanisms leading to functional hierarchy in neural systems.

## Author Summary

Functional hierarchy in neural systems, defined as the principle that complex entities may be segmented into simpler elements and that simple elements may be integrated into a complex entity, is a challenging area of study in neuroscience. Such a functional hierarchy may be thought of intuitively in two ways: as hierarchy in *space*, and as hierarchy in *time*. An example of hierarchy in space is visual information processing, where elemental information in narrow receptive fields is integrated into complex features of a visual image in a larger space. Hierarchy in time is exemplified by auditory information processing, where syllable-level information within a short time window is integrated into word-level information over a longer time window. Although extensive investigations have illuminated the neural mechanisms of spatial hierarchy, those governing temporal hierarchy are less clear. In the current study, we demonstrate that functional hierarchy can self-organize through multiple timescales in neural activity, without explicit spatial hierarchical structure. Our results suggest that multiple timescales are an essential factor leading to the emergence of functional hierarchy in neural systems. This work could contribute to providing clues regarding the puzzling observation of such hierarchy in the absence of spatial hierarchical structure.

## Introduction

Functional hierarchy, defined broadly as the principle that complex entities may be segmented into simpler elements and that simple elements may be integrated into a complex entity, is a ubiquitous feature of information processing in biological neural systems [1]–[4]. For example, in primary sensory areas such as VI and SI, the receptive field of neurons is relatively small, and these neurons respond to features of the stimulus that are simpler than those responded to by higher associative areas. Determining how these functional hierarchies are implemented in neural systems is a fundamental challenge in neuroscience.

The human motor control system is a representative example of a system with functional hierarchy. Humans acquire a number of skilled behaviors through the experience of repeatedly carrying out the same movements. Certain components of such movements, through repetitive experiences, are segmented into reusable elements referred to as “primitives”. In adapting to various situations, series of motor primitives are in turn also integrated into diverse sequential behavior. The idea underlying this basic process was proposed by Arbib in terms of “schema theory” [5], and has since been used as the basis for many studies (e.g. [6],[7]).

The action of drinking a cup of coffee, for example, may be broken down into a combination of motor primitives such as the motion of reaching for a cup on the table, and the motion of grasping the cup and bringing it to one's mouth. Ideally, these motor primitives should be represented in generalized manner, in the sense that the representation should be adaptive for differences in locations and in shapes of the cup. Primitives must also be flexible with respect to changes in the sequence of actions; for example, after grasping a cup, one sometimes brings the cup to one's mouth to drink, but one also sometimes takes the cup off the table to wash up. It is this adaptability (intra-primitive level) and flexibility (inter-primitive level) of primitives that allow humans to generate countless patterns of sequential behavior.

A number of biological observations suggest the existence of motor primitives. At the behavioral level, Thoroughman [8] for example showed that humans learn the dynamics of reaching motions through a flexible combination of movement elements. Sakai showed that, in visuomotor sequential learning, human subjects spontaneously segmented motor sequences into elementary movements [9]. At the level of animal muscle movement, Giszter [10], through observations of muscle movement in the frog's leg, found that there are a finite number of linearly combinable modules, organized in terms of muscle synergies on limbs. At the brain level, meanwhile, it has been shown that electrical stimulation in the primary motor and premotor cortex of the monkey brain evokes coordinated movements, such as reaching and grasping [11].

These observations strongly suggest that the diversity of behavior sequences in animals is made up of flexible combinations of reusable movement elements, i.e. motor primitives. What is not yet clear, however, is what underlying neural mechanisms govern the segmentation of continuous sensori-motor flows into primitives, and how series of primitives are combined into a variety of different behavior sequences.

To address this issue, we propose a neural network model for describing the neural mechanisms of segmentation and integration in continuous sensori-motor flows. This work can, as such, be seen as one possible neural implementation of schema theory. In experiments, the proposed network model was tested through the interaction of a humanoid robot with a physical environment, the robot requiring high-dimensional sensori-motor control. The robotics experiment is important when one considers the idea of the embodied mind by Varela [12], who explained that cognitive functions of neural systems emerge not only in the brain, but also in dynamic interactions between the physical body and the environment (see also a recent review [13]). This idea is also related to the so-called “synthetic approach” to neuroscience (or “robotic neuroscience”), an approach which has as its aim to extract essential mechanisms of neural systems using a variety of neuro-cognitive robotics experiments [14],[15].

**Figure 1:**
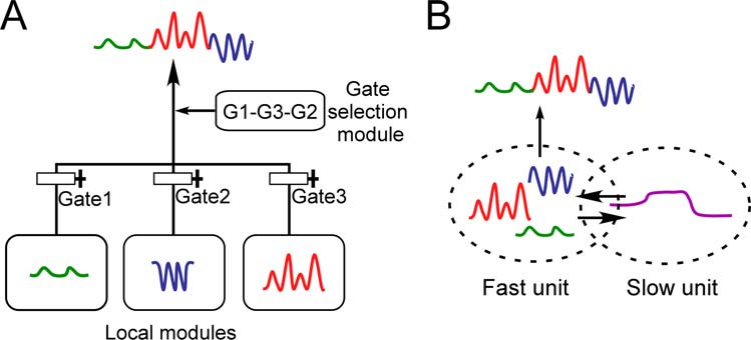
Schematic drawings of (A) local representation model and (B) multiple timescale model. (A) Curves colored red, blue, and green represent sensori-motor sequences corresponding to motor primitives. Output of the system consists of behavior sequences made up of combinations of these primitives. In the local representation model, functional hierarchy is realized through the use of explicit hierarchical structure, with local modules representing motor primitives in the lower level, and a higher module representing the order of motor primitives switched via additional mechanisms such as gate-selection. (B) In the multiple timescale model, primitives are represented by fast context units whose activity changes quickly, whereas sequences of primitives are represented by slow context units whose activity changes slowly.

There exist earlier studies on the computational modeling of functional hierarchy in sequences of motor primitives, representative examples being the “mixture of expert” model [16] and the “MOSAIC” model [17]. In these studies, functional hierarchy is realized through the use of explicit hierarchical structure, with local modules representing motor primitives in the lower level, and a higher module representing the order of motor primitives switched via additional mechanisms such as gate-selection (Figure 1A). We refer to this type of model as the “local representation” model.

There are a number of possible advantages to the local representation. First, the learning of one module would seem not to affect other modules. Second, based on this independence in the learning process, it would seem that increasing the number of local modules would lead to an increase in the number of acquirable primitives. An earlier study using multiple sensori-motor sequences, however, demonstrated that difficult problems arise in the local representation model as a result of its local nature [18]. Similarities in learned sensori-motor sequences create competition in the learning process between corresponding modules. Generalization requires similar patterns to be represented in the same module as the same primitive, even subtle differences exist in the treatment of sets of between such patterns. On the other hand, for the purposes of achieving “crisp” segmentation of sensory-motor flow, different patterns must be represented as separate primitives in distinct modules. This conflict between generalization and segmentation poses serious problems in the treatment of set of multiple sensori-motor sequences within which there are similarities and overlap. Due to the difficulty of this problem, it is not possible to increase the number of acquirable primitives simply by increasing the number of local modules [18]. In addition, due to the explicit hierarchical structure of the local representation, learning of the lower module (primitives) and learning of the higher module (sequences of primitives) have to be explicitly separated through subgoals arbitrarily set by the experimenter [15],[16].

In order to overcome difficulties associated with the local representation model, we introduce in the current study a different type of representation for functional hierarchy. The representation we use neither makes use of separate local modules to represent primitives, nor introduces explicit hierarchical structure to manipulate these primitives. Instead of setting up an explicit hierarchy, we attempt to realize the self-organization of a functional hierarchy by means of neural activity with multiple timescales. This functional hierarchy is made possible through the use of two distinct types of neurons, each with different temporal properties. The first type of neuron is the “fast” unit, whose activity changes quickly over the short term. The second type of neuron is the “slow” unit, whose activity changes over the long term (Figure 1B).

The idea that multiple timescales may carry advantages for neural systems in interacting with complex environments is intuitively understandable. Indeed, the importance of multiple timescales in neural systems has been emphasized in a number of earlier studies from various different fields. For example, at the level of behavior, it has been shown that the process of acquiring motor skills develops through multiple timescales [19],[20]. Biological observations on motor adaptation, such as for example saccade adaptation and force field adaptation, likewise suggest that these processes involve distinct subsystems with differing timescales [21],[22]. At the level of neural synchrony, meanwhile, it is thought that differing timescales in neural synchrony are involved at different levels of information processing, such as for example in local and global interactions of brain regions [23],[24]. These previous studies strongly suggest the possibility that multiple timescales may be essential for the emergence of functional hierarchy in neural systems.

At the neuron level, the use of timescale variation has also been proposed as a means of representing different levels of functionality. In a study of auditory perception, for example, Poeppel [25] hypothesized that different temporal integration windows in neural activities correspond to a perceptual hierarchy between formant transition level and syllable level. In a study of an evolutional neural network model using a mobile robot, Nolfi [26] showed that a model with differing temporal integration windows is superior to the normal model in cases in which the robot is required to achieve two different tasks: collision avoidance, which requires short-term sensori-motor control, and self-localization, which requires long-term sensory integration. Furthermore, Paine [27] showed that, using a similar evolutional neural network model with a mobile robot, it was possible to achieve hierarchical functionality of motor primitives (wall avoidance) and execution of a given sequence of primitives (global goals) through a particular constraint on neural connectivity. In this model, one part of the network evolved so as to be responsible for primitives with fast dynamics, whereas another part of the network evolved so as to be responsible for sequences of primitives with slower dynamics. Paine's study is similar to the current study in that, in the functional hierarchy between motor primitives and behavior sequences, no separate local modules are used to represent primitives, and neither is any explicit hierarchical structure used to manipulate these primitives.

In the current study, however, our focus is on studying the impact to neural activity of multiple timescales. Unlike the earlier study by Paine, in which multiple timescales evolved as a result of an explicit requirement for different levels of functionality, in the current study we investigate whether functional hierarchy can self-organize through the imposition of constraints on timescales of the network. The proposed model will show that, through repetitive execution of skilled behavioral tasks, continuous sensori-motor flows are segmented into reusable motor primitives (adaptable to differences in location), and segmented primitives are flexibly integrated into new behavior sequences. The model does this without setting up an explicit sub-goal or functions such as gate-selection for manipulating primitives in the lower module, deriving this functional hierarchy instead through the use of distinct types of neurons, each with different temporal properties.

The main focus of the current study is on the question of how temporal behavior sequences can arise from neural dynamics. Thus we chose a dynamical systems approach [28] using a neural network model rather than a statistical model, the latter of which is often used as a powerful tool for studying mechanisms of neural systems [29]–[31]. Among dynamical systems models, the use of physiologically detailed models with spiking neurons has become popular in explaining accumulated neurophysiological findings [32]–[34]. It is nonetheless still difficult to reproduce diverse sequential behavior in robots starting at the level of models with spiking neurons. In the current study, in order to mediate between the conceptual level of schema theory and the physiologically detailed level of models using spiking neurons, we propose a macro-level neural dynamics model.

The main component of the current model is a continuous time recurrent neural network (RNN). Thanks to its capacity to preserve the internal state, which enables it to reproduce complex dynamics, the RNN is often used for modeling temporal sequence learning [35]–[37]. The continuous time RNN (CTRNN) is a type of RNN which implements a feature of biological neurons, namely that the activities of neurons are determined not only by current synaptic inputs but also by the past history of neural states. Due to this characteristic, according to which activation changes continuously, the CTRNN is superior to discrete time RNN models in modeling mechanisms for producing continuous sensori-motor sequences [38],[39].

The model of neurons is a conventional firing rate model, in which each unit's activity represents the average firing rate over a group of neurons. Spatio-temporal patterns of behavior arise from dynamics of neural activities through neural connectivity. The CTRNN is as such considered to emulate characteristic features of actual neural systems, and the current model is considered consistent at the level of the macro-level mechanisms of biological neural systems. For this reason, consistency in physiological details, such as features of neural activity at the level of individual neurons and characteristics of individual synapses, are not considered in detail. It is not our intention in the current study to map directly between model components and actual brain structures. Possible implications to biology of the current results were discussed only at an abstract level, in terms of the model employed in the current study.

## Results

### Task Design

**Figure 2:**
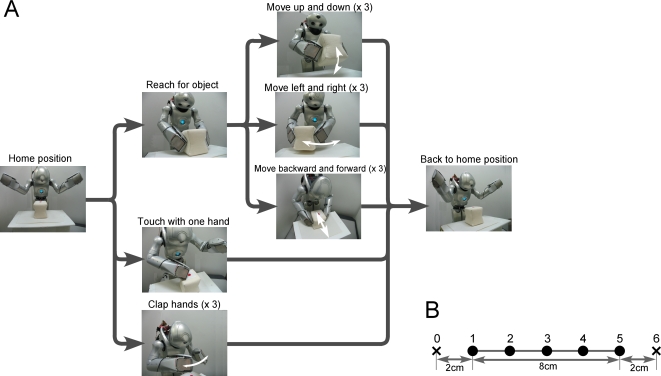
Task design. (A) A humanoid robot was fixed to a stand. In front of the robot, a workbench was set up, and a cubic object (approximately 9×9×9 cm) was placed on the workbench to serve as the goal object. The task for the robot was to autonomously generate five different types of behavior: (1) move the object up and down three times, (2) move the object left and right three times, (3) move the object backward and forward three times, (4) touch the object with one hand, and (5) clap hands three times. For each behavior, the robot began from the home position and ended at the same home position. (B) For each behavior other than the clapping hands task, the object was located at five different positions (positions 1–5). Since the clapping hands behavior was independent of the location of the object, the object was located at the center of the workbench (position 3) and was never moved for this task.

A small humanoid robot was used in the role of a physical body interacting with actual environment. A workbench was set up in front of the robot, and a cubic object (approximately 9×9×9 cm) placed on the workbench served as the goal object. The task for the robot was to autonomously reproduce five different types of learned behavior (referred to as the “basic” behavior patterns): (1) move the object up and down three times, (2) move the object left and right three times, (3) move the object backward and forward three times, (4) touch the object with one hand and (5) clap hands three times. For each behavior, the robot's task began from the same home position and ended with the home position (Figure 2A).

As shown in Figure 2A, task trajectories had a temporal structure which could be described by a path of state transitions with branching, although there was no explicit trigger for branching. From the home position, trajectories branched three ways, each corresponding to different actions: reaching for the object, touching with a single hand, and clapping. After reaching for the object, trajectories again branched three ways for different possible actions: moving the object up and down, moving it left and right, and moving it backward and forward. Even with repetitive movement such as moving the object up and down, there was potential branching in the possibility of either repeating the up-down movement one more time, or going back to the home position. This temporal structure of task sequences was characterized by the presence of multiple timescales, with sensori-motor flows changing rapidly over the short term and task sequences following a state transition structure with branching over the long term.

### System Overview

**Figure 3:**
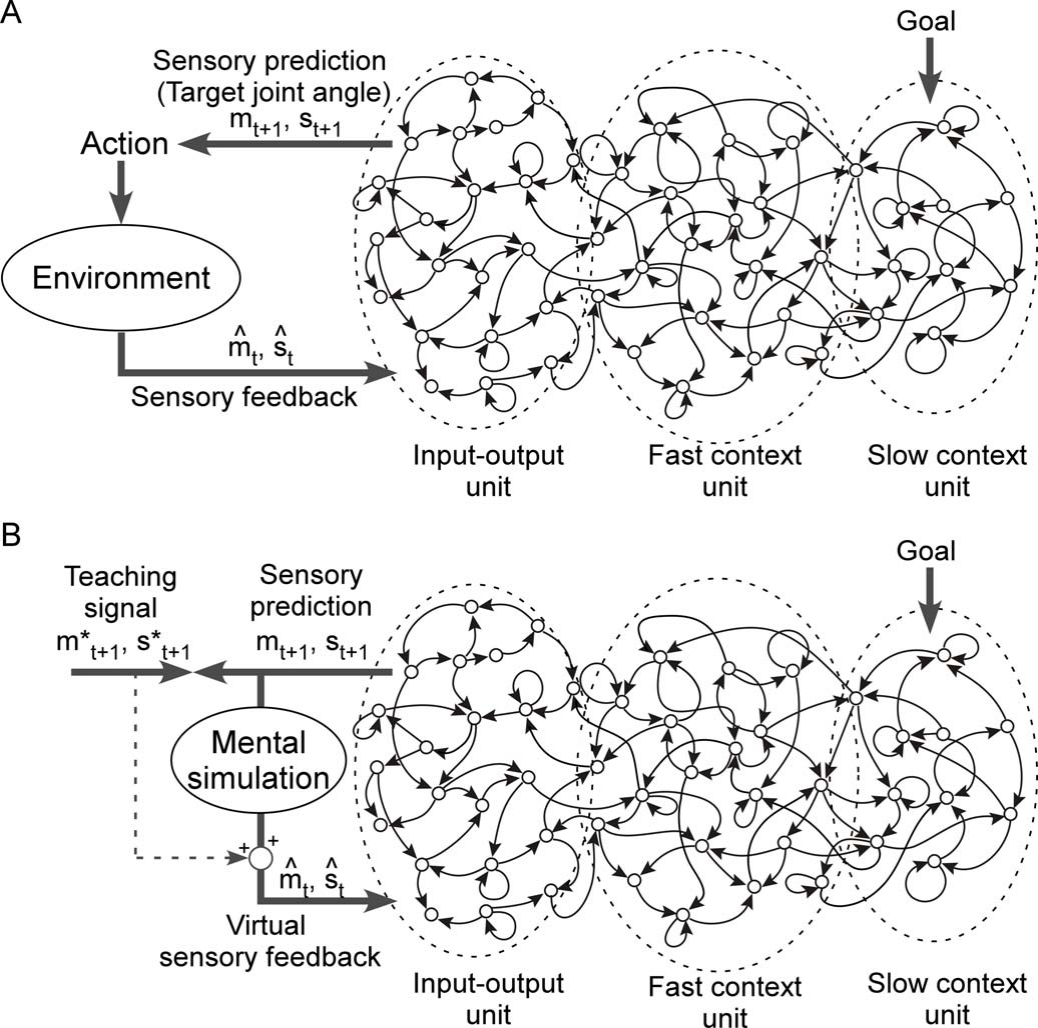
System overview. (A) Action generation mode. Inputs to the system were the proprioception *mˆ* *_t_* and the vision sense *ŝ* *_t_*. Based on the current *mˆ* *_t_* and *ŝ* *_t_* the system generated predictions of proprioception *m_t_*_+1_ and the vision sense *s_t_*_+1_ for the next time step. This prediction of the proprioception *m_t_*_+1_ was sent to the robot in the form of target joint angles, which acted as motor commands for the robot in generating movements and interacting with the physical environment. Changes in the environment were sent back to the system as sensory feedback. The main components of the system were modeled by the CTRNN, which is made up of input-output units and context units. Context units were divided into two groups based on the value of time constant *τ*: a group of fast context units (*τ* = 5) and a group of slow context units (*τ* = 70). Every unit of the CTRNN is connected to every other unit, including itself, with the exception of input units which do not have a direct connection to the slow context units (see Method). (B) Training mode. In the training process, the network generates behavior sequences based on the synaptic weights at a certain moment during the learning process. Synaptic weights are updated based on the error between generated predictions (*m_t_*_+1_, *s_t_*_+1_) and the teaching signals (m*_t__+1_, s*_t__+1_). In training mode, the robot did not interact with physical environment. Instead of actual sensory feedback, predicted proprioception and vision served the input for the following time step (mental simulation). Through this mental simulation process, the network was able to autonomously reproduce behavior sequences without producing actual movements. In addition to virtual sensory feedback, in order to accelerate convergence, a small amount of the teaching signal of the previous time step m*_t__+1_, s*_t__+1_ was also mixed into *m_t_*_+1_, *s_t_*_+1_ (see Method for details). Both in the generation mode and training mode, initial state of the slow context units was set according to the task goal.

Inputs to the system were the proprioception *mˆ* *_t_* (8 dimensional vector representing the angles of arm joints) and the vision sense *ŝ* *_t_* (2 dimensional vector representing object position) (Figure 3). Based on the current *mˆ* *_t_* and *ŝ* *_t_*, the system generated predictions of proprioception *m_t_*_+1_ and the vision sense *s_t_*_+1_ for the next time step. This prediction of the proprioception *m_t_*_+1_ was sent to the robot in the form of target joint angles, which acted as motor commands for the robot in generating movements and interacting with the physical environment. This process, in which values for the motor torque are computed from the desired state, is considered, at a computational level, to correspond to the inverse model [40],[41]. This inverse computation process was preprogrammed in the current system within the robot control system. Changes in the environment, including changes in object position and changes in the actual position of joints, were sent back to the system as sensory feedback.

The main component of the system modeled by the CTRNN received two different modality inputs, proprioceptive somato-sensory input and vision input. These different modality sensations came together in the CTRNN to generate predictions of the future state. These predictions were made possible by the capacity of the CTRNN to preserve the internal state, which enables it to reproduce complex dynamics. This type of computation, in which the next sensory state is predicted from the current state, is considered to correspond to the forward model [40]–[43].

In the CTRNN, proprioception and vision inputs were sparsely encoded in the form of a population coding with the preserving topology of the input space (see Method for details). This topology preserving sparse encoding of sensori-motor trajectories reduced overlap between sensori-motor sequences and improved the learning capacity of the CTRNN.

A conventional firing rate model, in which each unit's activity represents the average firing rate over a group of neurons, was used to model neurons in the CTRNN. In addition, every unit's membrane potential was assumed to be influenced not only by current synaptic inputs, but also by their previous state. This characteristic is described by the following differential equation, which uses a parameter *τ* referred to as the time constant:
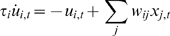
(1)where *u_i,t_* is the membrane potential, *x_i,t_* is the neural state of the *i*th unit, and *w_ij_* is synaptic weight from the *j*th unit to the *i*th unit. The second term of Equation 1 corresponds to synaptic inputs to the *i*th unit. The time constant *τ* is defined as the decay rate of the unit's membrane potential, analogous to the leak current of membrane potential in real neurons. One might consider this decay rate to correspond to an integrating time window of the neuron, in the sense that the decay rate indicates the degree to which the earlier history of synaptic inputs affects the current state. When the *τ* value of a unit is large, the activation of the unit changes slowly, because the internal state potential is strongly affected by the history of the unit's potential. On the other hand, when the *τ* value of a unit is small, the effect of the history of the unit's potential is also small, and thus it is possible for activation of the unit to change quickly.

The network that was used in the current model consisted of input-output and non-input-output units, the latter referred to as context units. Context units were divided into two groups based on the value of time constant *τ*. The first group consisted of fast context units with small time constant (*τ* = 5) whose activity changed quickly, whereas the second group consisted of slow context unit with a large time constant (*τ* = 70) whose activity, in contrast, changed much more slowly. Among the input-output units, units corresponding to proprioception and units corresponding to vision were not connected to each other. In addition, inputs were also not directly connected to slow context units.

### Training

In order to obtain a teaching signal, the experimenter guided both hands of the robot along the trajectory of the goal action. As the robot hands were guided along the trajectory, encoder values of each joint were recorded, and recorded sensori-motor trajectories were used as teaching sequences. For each behavior task other than the clapping of hands, the object was located in five different positions (position 1 to position 5 in Figure 2B). Since the action of clapping hands was independent of object location, the object was always located at the center of the workbench for this task (position 3).

The objective of learning was to find optimal values of connective weights minimizing the error between teaching sequences and model outputs. At the beginning of training, synaptic weights of the network were set randomly, resulting in the network generating random sequences. Synaptic weights were modified based on the error between teaching signals and generated sequences. After many repetitions of this process, the error between teaching sequences and model outputs eventually reached a minimum level.

This training process was conducted in an off-line manner, in the sense that the prediction of the sensory-motor trajectories were generated by means of so-called closed-loop operations (Figure 3B) in which the current prediction of the proprioception and vision state are used as input for the next time step.

Nishimoto [44] demonstrated that the RNN can learn to generate multiple sequences starting from different initial states through an association between initial states and corresponding sequences. Utilizing this characteristic of initial sensitivity, the CTRNN was trained to generate multiple behavior sequences through the selection of corresponding initial states, defined by the experimenter.

In the proposed model, a network was trained by means of supervised learning using teaching sequences obtained through tutoring by the experimenter. The conventional back-propagation through time (BPTT) algorithm was used for learning of the model network [45]. In the current study, the BPTT was used not for mimicking the learning process of biological neural systems, but rather as a general learning rule. Results obtained reflect characteristic features of the proposed network architecture, and not of the learning algorithm. Similar results could be obtained using a different learning algorithm, such as for example the biologically plausible algorithm proposed by Seung's group [46],[47], a kind of reinforcement learning.

### Action Generation in Physical Environment and Mental Simulation

Through the training process, the network learned to predict sensory feedback for the next time step. This prediction of sensory feedback was treated as a target joint angle, and was sent to the robot. Following this target joint angle, the robot was in turn able to reproduce learned movements even in a physical environment. This physical environment included fluctuations that were unavoidable given the conditions of the experiment, such as for example fluctuations of sensory inputs resulting from imprecision in motor control, as well as fluctuations resulting from the instability of light on vision sensors. Fluctuations were also caused by unstable positioning of the object resulting from nonlinear friction between the object, the robot arm, and the workbench.

Moreover, by using the prediction of sensory feedback as input to the next time step (closed-loop generation), the network was able to autonomously generate sensori-motor trajectories without producing actual movements. This process of closed-loop generation was treated as corresponding to the mental simulation of actions [48],[49].

### Performance of Robot

Five learning trials were conducted with different initial values for synaptic weights. The BPTT was conducted over 5000 iterations, with optimal performance weights taken as the set of weight values for which error was minimized. Model networks with these optimal weights were tested through the interaction of the robot with a physical environment. Learning error and performance of the robot, for all types of behavior and for all different object positions, is summarized in the Table 1. Interacting with the physical environment, the robot was able to nearly perfectly reproduce learned behavior, and also successfully adapted to differences in the location of objects. Success or failure was judged according to criteria described later in this paper (see Method for details).

**Table 1:** Learning error and robot performance for the basic pattern training.

	Error	Robot Performance (%)
Trial 1	0.475	100
Trial 2	0.488	100
Trial 3	0.469	95.24
Trial 4	0.469	100
Trial 5	0.464	100
Mean (SD)	0.473 (0.009)	99.05 (2.13)

Five learning trials were carried out with different initial synaptic weight values. Each behavior of the robot was tested in every position. Performance was scored in terms of the success rate over all tasks. Success or failure in the robot performance test was judged according to criteria described in Method.

**Figure 4:**
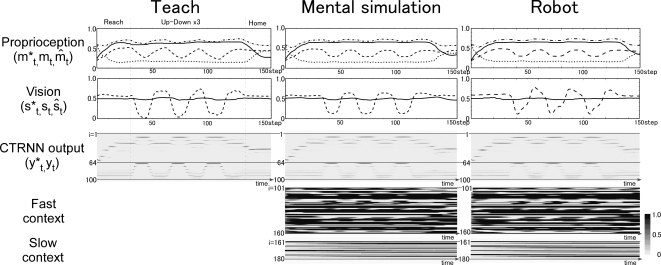
Example of behavior sequence for up-down behavior. Proprioception (first row), vision (second row), sparsely encoded RNN activation (third row), fast and slow context activation (forth and fifth row) of teaching signal (left column), mental simulation of trained network (center column) and actual sensory feedback in physical environment (right column) during up-down behavior at position 3 are shown. In proprioception, 4 out of a total of 8 dimensions were plotted (full line: left arm pronation, dashed: left elbow flexion, dot-dash-dot-dash: right shoulder flexion, dotted: right arm pronation). In the case of vision, two lines correspond to the relative position of the object (full line: X-axis, dashed line: Y-axis). Values for proprioception and vision were mapped to the range from 0.0 to 1.0. CTRNN outputs are sparsely encoded. Both in CTRNN outputs and context activation, the y axis of the graph corresponds to each unit from among the output units and context units. A long sideways rectangle thus indicates the activity of a single neuron over many time steps. The first 64 units of output correspond to proprioception and the last 36 units of output correspond to vision. Colors of rectangles indicate activation level, as indicated in the color bar at the lower right. Reach: reach for the object, UD: up-down, Home: return to the home position.

**Figure 5:**
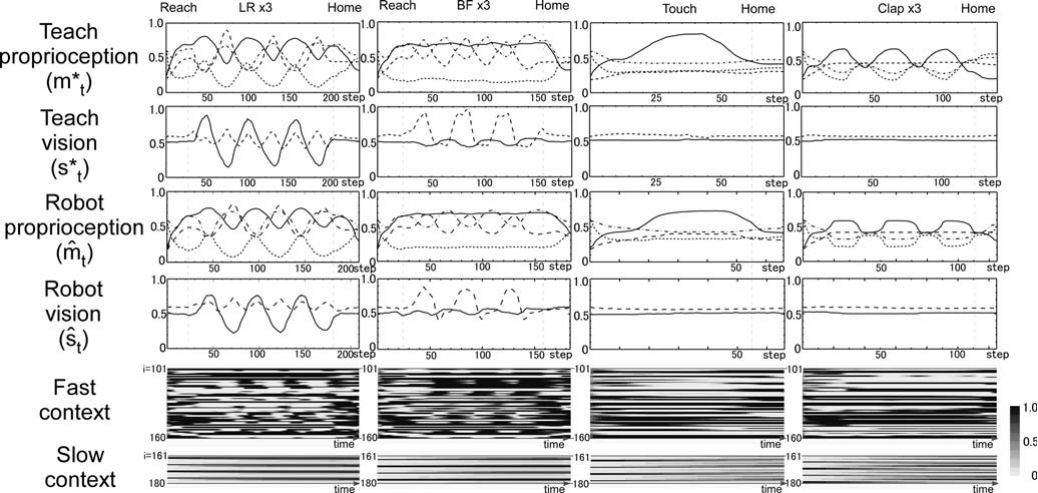
Example of behavior sequences for other basic behavior. Proprioception, vision, fast and slow context activation of teaching signal and actual values in physical environment during left-right (LR: first column), backward-forward (BF: second column) touch with single hand (Touch: third column) and clapping hands (Clap: fourth column) behavior at position 3 are shown. Correspondences for line types in each graph are the same as in Figure 4. Reach: reach to the object, Home: return to the home position.

Figure 4 and Figure 5 illustrate examples of sensori-motor sequences, as well as examples of teaching signals and trained model network interacting with a physical environment through the body of the robot. Figure 4 also includes examples sequences generated by mental simulation. Both in mental simulation and in the context of the robot interacting with a physical environment, the trained network reproduced target behavior sequence successfully.

### Representations in the Fast and Slow Context Unit

When the robot generated repetitive movements such as moving the object up and down three times, repetitions of similar patterns were observed in activities of the fast context units. The slow context units, in contrast, changed gradually, and no such repetitive patterns were observed (Figures 4 and 5). Changes in the value of slow context units seemed to drive switching between movements, for example between repetitive movements and the action of going back to the home position. These patterns in the activation of context units suggest that the fast context units encoded reusable movement segments (“primitives”), whereas the slow context units encoded the switching between these primitives.

In order to confirm this hypothesis, internal network representations for each pattern of behavior were investigated by analyzing the activation of context units for different behavior and for different positions. For every behavior at every position, context unit activation values were recorded as sequences of sixty dimensional vectors (fast context) and twenty dimensional vectors (slow context). The dimensionality of these multidimensional data sets was reduced using principal component analysis (PCA).

**Figure 6:**
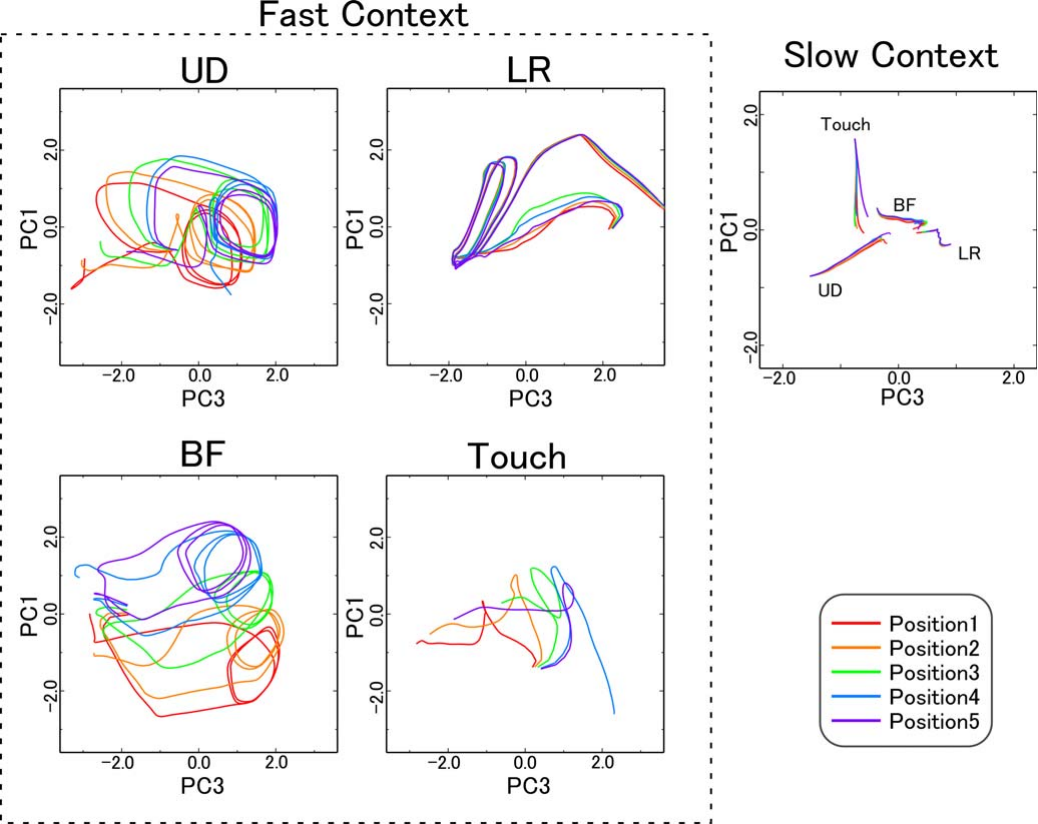
Changes in context state space associated with changes in object position. Changes of context activation during each behavior at every position are shown in a 2 dimensional space based on the results of PCA analysis. The four graphs on the left side and single graph on the right side correspond to fast context activities and slow context activities, respectively. State changes of the fast context units for each behavior exhibit a particular structure which shifts with the object position. On the other hand, activity of the slow context units for a particular behavioral task exhibited very little location-dependent variation. UD: up-down, LR: left-right, BF: backward-forward and Touch: touch with single hand.

In order to visualize changes of state in the network during execution of behavioral tasks, two principal components of context unit activation values were plotted in Figure 6 for every behavior and at every position. The clapping hand behavior was not plotted as this behavior was independent of object position.

Activity of the slow context units exhibited very little location-dependent variation, and no patterns corresponding to repetitive movements were observed. On the other hand, in the fast context units, trajectories for each behavior exhibited a particular structure which shifted with the object position. This representation of behavior sequences in the state space of fast context units reflected characteristic features of the current tasks: the bulk of the task sequences consisted of cyclic patterns (e.g. repetitions of up-down motion, left-right motion, and backward-forward motion), and the position of the object acted on by the robot shifted along a one-dimensional axis. In the up-down behavior, for example, closed curves corresponded to cyclic patterns of up-and-down motion, and shifts of these curves corresponded to one-dimensional shifts in object location.

These observations suggest that functional hierarchy of primitives and sequence of primitives was self-organized in the model network. That is, in the task behavior sequences, movements that appeared repeatedly (e.g. cyclic patterns) were segmented into reusable “primitives”. These primitives were represented in fast context dynamics in a form that was generalized across object locations. On the other hand, the slow context units appeared to be more abstract in nature, representing sequences of primitives in a way that was independent of the object location.

### Additional Training of Novel Primitive Combinations

From the hypothesis that fast context units and slow context units encode, respectively, motor primitives and sequences of primitives, one would anticipate that novel combinations of primitives would be generated only by altering the activity of the slow context units. In order to test this idea, the network was trained to additionally generate novel sequences of behavior assembled out of new combinations of primitives. During the additional training, only connections of the slow context units were allowed to change, weights of the other units remaining fixed at the values that were set through the basic training.

In the additional training, the robot was required to (a) move the object up and down three times, then move the object left and right three times and go back to the home position, and to (b) move the object backward and forward three times, then touch the object with one hand and go back to the home position.

**Figure 7:**
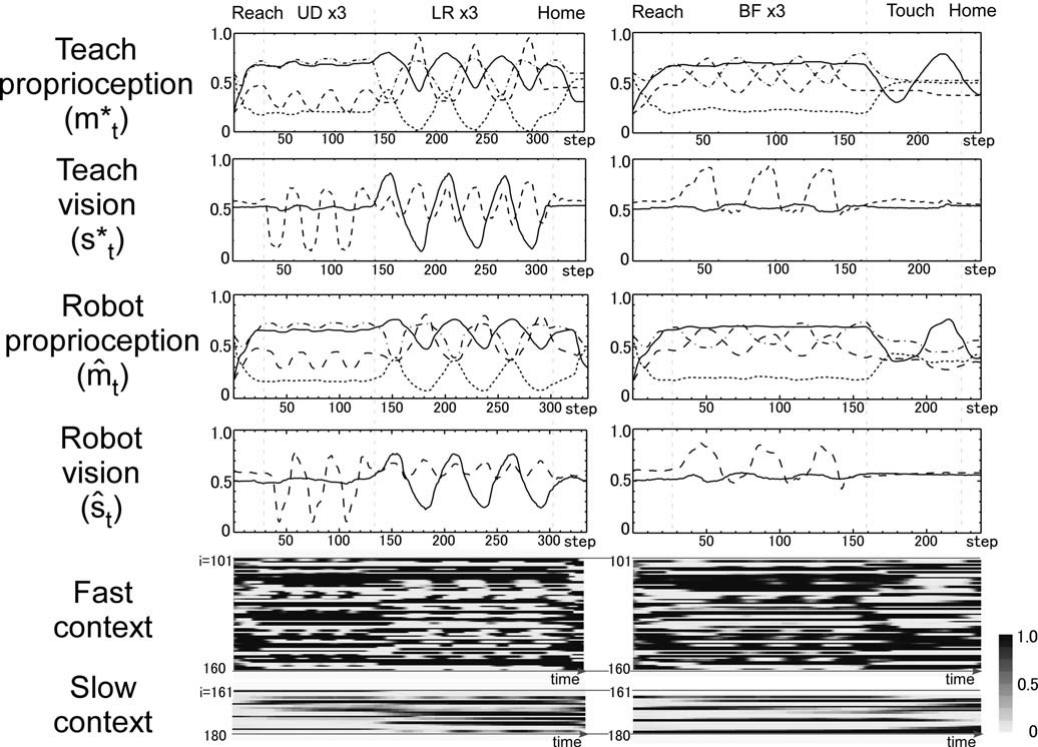
Example of behavior sequence for novel combinations of motor primitives. Proprioception, vision, and fast and slow context activation values of the teaching signal, as well as actual values in physical environment, are shown for two novel behaviors at position 3. The first behavior (left column) consists of moving the object up and down three times, then moving the object left and right three times, and finally returning to the home position. The second behavior (right column) consists of moving the object backward and forward three times and then touching the object with one hand, and finally returning to the home position. Correspondences for line types in each graph are same as in Figure 4. UD: up-down, LR: left-right, BF: backward-forward and Touch: touch with single hand. Reach: reach for the object, Home: return to the home position.

**Figure 8:**
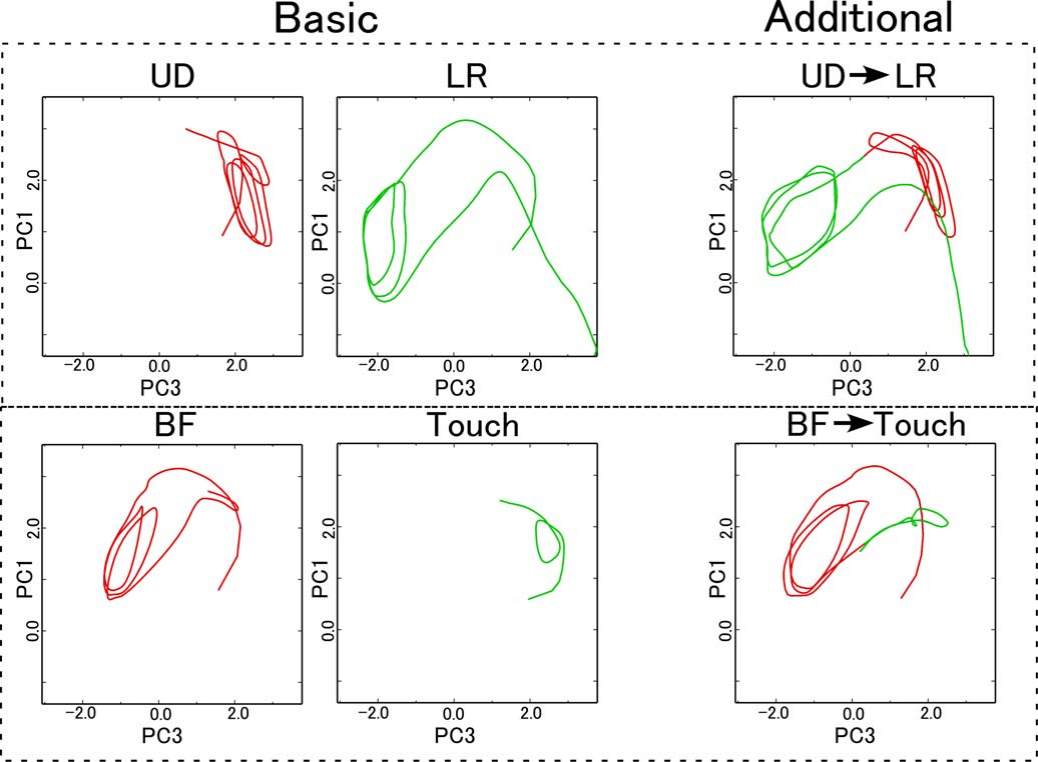
Primitive representations in fast context units before and after additional training. Changes of context activation during each movement before and after additional training are visualized in a 2 dimensional space based on the results of PCA analysis (plotted only for position 3). The four graphs on the left side and two graphs on the right side correspond to representations before and after additional training, respectively. The first and second movements in the novel sequences learned through additional training are colored red (UD and BF) and green (LR and Touch), respectively. The structure of representations corresponding to each primitive were preserved even after additional training, indicating that motor primitives were represented in dynamics of fast context units, with novel behavior sequences constructed out of combinations of these primitives. UD: up-down, LR: left-right, BF: backward-forward and Touch: touch with single hand behavior.

Through training, the robot was able to reproduce perfectly the novel behavior sequences generalized across object locations, and also managed to successfully interact with the physical environment. Figure 7 displays examples of sensori-motor sequences as well as of neural activities of the teaching signal and trained model network interacting with the physical environment. Context unit activations corresponding to the same behavior were observed to be similar both in the first basic behavior training and in the additional training. Context unit activation values corresponding to left-and-right movement in basic behavior training, for example, were almost identical to context unit activation values corresponding to left-and-right movement in the novel sequences used in the additional training. In order to verify this observation, as in the previous section, PCA was again conducted for the fast context unit activation values during the execution of novel sequences of behavior. Figure 8 shows examples of changes in the states of context units for two cases: during the execution of four basic behavioral patterns following basic pattern training, and during the execution of novel behavior sequences following additional learning.

In the graphs of activation values both in basic pattern training and in additional training, representations for each motor primitive were preserved. For example, the cyclic pattern corresponding to the up-and-down movement in basic learning was preserved in the novel behavior sequence of the additional training (red line in upper graphs of Figure 8).

These results indicate the role of functional differentiation in the current model: motor primitives, such as reaching for the object, moving the object up and down, and moving the object left and right, were represented in the dynamics of fast context units, whereas activities of the slow context units represented switching of these primitives. By changing activities of slow context units, segmented primitives moreover were integrated into new behavior sequences by combining them in different orders.

### Significance of Multiple Timescales

**Figure 9:**
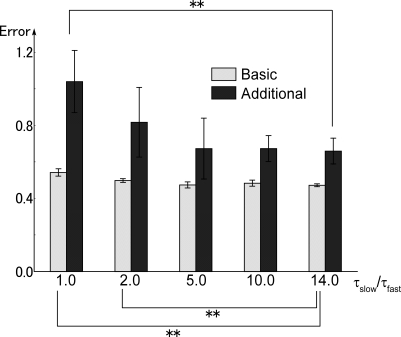
Effects of multiple timescales. Learning error for basic pattern and novel pattern training for various slow context time constant values are shown. Differences in timescale are described by the ratio of *τ* values in the fast and slow context units (*τ*-slow/*τ*-fast). Bars in the graph correspond to mean values over 5 learning trials for each parameter setting. Error bars indicate the degree of standard deviation. Asterisks indicate significant differences in mean values between the standard setting (*τ*-ratio = 14.0) and other settings. The significance of these differences was examined using a randomized test. Both in basic pattern training and in additional training, performance for the case of small *τ*-ratio was significantly worse than the standard setting. These results suggest that multiple timescales in the fast and slow context units was an essential factor leading to the emergence of hierarchical functional differentiation.

In order to investigate the impact of multiple timescales on hierarchical functional differentiation, performance of the model was tested while changing the value of the time constant parameter *τ* in the slow context units, while the value of *τ* in the fast context units was held fixed at 5. Difference in timescales was described in terms of the ratio of *τ* values in the fast and slow context units (*τ*-slow/*τ*-fast). For each value of this *τ*-ratio, five trials were conducted for both the basic training of five behavior patterns, and for the training of novel patterns. Mean values of the learning error for all *τ*-ratio settings are presented in Figure 9. The significance of differences between the standard setting (*τ*-ratio = 14.0) and other settings was examined using a randomized test.

In basic pattern training, performance for small *τ*-ratios (*τ*-slow/*τ*-fast values of 1.0 and 2.0) was significantly (p<0.01) worse than for the standard setting. In the additional training, where the network was required to reconstruct longer, novel behavior sequences from combinations of primitives as represented by fast context units, difference in error appeared to be much larger than in the basic training. In the case when the *τ*-ratio was set to 1.0, so that there was no difference in time constant between fast and slow context units, performance was significantly (p<0.01) worse than performance with the standard setting. These results suggest that multiple timescales in context units was an essential factor leading to the emergence of hierarchical functional differentiation. Specifically, for cases in which the value of the *τ*-ratio was more than 5.0, performance of the model was significantly higher than in cases of lower *τ*-ratio values. One possible explanation for this observation is that the optimal ratio between *τ*-slow and *τ*-fast (*τ*-slow/*τ*-fast = 5.0) may correspond to the ratio between the total length (in time steps) of the task sequence (from the home position back to the home position) and the length of each primitive, i.e. the ratio (total length/primitive length).

## Discussion

### Model Mechanisms

The capacity of the CTRNN used in this study to capture forward dynamics results from the self-organization of context state dynamics associated with continuous sensori-motor flows. One of the characteristics of CTRNN essential in learning and reproducing multiple patterns of sensori-motor sequences is their initial sensitivity [44]. The CTRNN used in this study were able to represent multiple sensori-motor sequences through associations between various initial states and internal dynamics of context units. Initial states associated with particular behavior sequences can be thought of as corresponding to goal information for motor control systems.

In the current study, initial states of the slow context units were set in such a way as to specify task goals. Initial states were set at different values for different target behavior sequences, regardless of the location of the object, which was situated in five different positions. Other than initial states of the slow context units, all parameters, including initial states of the input-output units and of the fast context units, were held constant for all task behaviors. This means, in other words, that if initial states of the slow context units had not been set, the network would not have been able to produce multiple behavior sequences.

The CTRNN was trained to reproduce multiple sensori-motor sequences through an association between the goal of a given task and the internal dynamics of the context units. Associating initial states with multiple different sensory-motor sequences, each taking the form of state transition structures with branching, is however not straightforward. While sensori-motor states in such behavior sequences change rapidly over short timescales, the same sequences take on the form of a state transition structure with frequent branching over longer timescales. This trajectory structure gives rise to a conflict in the selection of suitable time properties for the context units [39]. A large time constant *τ* enables context units to develop dynamics that change slowly, necessary in preserving goal information over long trajectories with frequent branching. In order for context units to capture changes in sensori-motor trajectories that occur over short timescales, however, a small time constant *τ* is needed.

This conflict in time properties of the network places a demand on context units to operate at multiple timescales. In order to satisfy this demand, we introduced a “multiple timescale RNN (MTRNN)” in which a network is made up of two different types of context units, each type with its own distinct time constant: large *τ* (slow context) and small *τ* (fast context). In the current set of tasks, the ratio of the time constants in context units (*τ*-slow/*τ*-fast) played a crucial role in the emergence of hierarchical functional differentiation, where units with small and large time constants corresponded, respectively, to primitives and combinations of primitives in sensori-motor sequences.

Through the process of training, fast context units develop short-timescale dynamics corresponding to changes in sensori-motor state. However, due to the short timescale of these dynamics, it is difficult for fast context units to preserve goal information; this goal information is essential in selecting appropriate branches along a trajectory toward the target behavior sequence. It is thus difficult, based on the dynamics of fast context units, to make predictions regarding sensori-motor trajectories at branching points, particularly when the branching point in question is far from the start of the behavior sequence.

As a result of this unpredictability at branching points, dynamics of the fast context units were segmented into behavioral elements, corresponding to primitives, extending from one branching point to the next. Prediction error resulting from the unpredictable nature of the dynamics of fast context units, meanwhile, drove the development of dynamics in the slow context units; the dynamics of these slow context units in turn triggered branch selection while preserving goal information. Through these mechanisms of self-organization, continuous sensori-motor flows of skilled behavior were segmented into reusable primitives.

Functional differentiation between slow context units and fast context units was confirmed in an analysis of the structure of context dynamics. As shown in Figure 6, behavior was represented in the slow context units in an abstract manner, in the sense that activity of the slow context units for a particular behavioral task exhibited very little location-dependent variation. Patterns of activity in the fast context units, in contrast, shifted with the position of the object, while at the same time preserving the trajectory shape particular to each behavior pattern. This indicates that the representation of primitives in the network was expressed through the dynamics of fast context units, in a way that was generalized across object locations. It was thus possible for the network, simply by shifting the activity of fast context units in accordance with sensory feedback, to adapt primitives in such a way as to accommodate differences in object location. As shown in Figure 8, these primitives were moreover successfully integrated into novel sequences of behavior, within which primitives were flexibly modified and assembled in various different orderings. This adaptivity (intra-primitive level) and flexibility (inter-primitive level) of primitives enabled the network to produce various patterns of sequential behavior.

There are two other factors, other than multiple timescales, which may be involved in the emergence of functional hierarchy in this study. The first factor is the method by which initial states are set. In order to specify task goals, initial states were set in the experiments in this study at values corresponding to different target behavior sequences, without relation to the location of the object. This position-independent “binding” of behavior may enhance the capability for achieving a generalized representation of behavior, and as such may affect the development of abstract representation in the slow context units. An alternative method, by which the setting of initial states is self-determined through a learning process, was demonstrated by our group recently in separate study [39]; in this context, initial values which correspond to the same behavior are very close to each other in the state space of initial values. This process, however, requires fine tuning of parameters in balancing, for example, the learning rate for initial states and the learning rate for connective weights.

In addition to the selection of initial state values, another consideration which may affect the emergence of hierarchy is the fact that information about task goals was given as initial states only for the slow context units, and not for the fast context units. The potential effect of this approach is that representations for task goals may not develop significantly in the fast context units without setting initial states in those units. The setting of such initial states, however, does not assure functional hierarchy, given that there is still a possibility that information corresponding to primitives could be mixed into the dynamics of slow context units.

The second possible factor determining the emergence of hierarchy is the way in which connections are constrained, in particular the fact that slow context units do not directly interact with input-output units. Due to this constraint, external input signals that change over short timescales do not directly affect the dynamics of slow context units, the same dynamics that carry goal information. This disconnect between goal information and external inputs is similar to the “bottle-neck” in Paine's work [27], where neurons of the higher module, which carry information about the task goal, interact with external inputs of the lower module only through a particular class of neurons referred to as bottle-neck neurons. In Paine's study, it was shown that functional hierarchy emerged more readily in the case of a network with a bottle-neck than in a network without a bottle-neck. The fast context units constraining information flow in the current model network do not constitute a “bottle-neck” in a literal sense of the word, but are more suitable referred to as “hub” nodes, considered to play an important role in the coordination of information flow in neural systems [50],[51]. The constraint on information flow may as such also help the network in realizing functional differentiation between fast and slow context units.

On the other hand, parameter analysis of time constant values in the current study indicated that performance of the model was significantly worse in the absence of differing timescales between the fast and slow context units, despite the fact that the method for setting initial states, as well as constraints on information flows, were the same across the whole network. Without multiple timescales, it is natural to expect that representations of primitives in each unit type would mix together and interfere with one another, making the production of novel combinations of primitives through the manipulation of slow context units impossible. These considerations suggest that, in the current model, the presence of multiple timescales in fast and slow context units is essential for the emergence of hierarchical functional differentiation between the level of primitives and that of sequences of primitives.

### Realization of Functional Hierarchy in Motor Control Systems

In biological studies of human and primate motor control systems, it is thought that cortical motor areas may be organized in a hierarchical manner [52],[53]. The activity of neurons in the primary motor cortex (MI), for example, is thought to be responsible for relatively low-level motor control in actions such as joint rotations and muscle forces [54],[55]. Neurons in the premotor cortex (PM), meanwhile, are thought to be involved in higher levels of motor control, such as for example preparation for movement [56],[57], specific action “vocabulary” (e.g. grasping, holding, and tearing, which correspond to motor primitives) [52],[58], and decisions in action selection [59],[60]. Finally, the supplementary motor area (SMA) is considered to play a role in controlling sequences of actions [61],[62]. Based on this organization, one would expect to observe hierarchical structure in anatomical connections of the SMA-PM-MI corresponding to functional hierarchy in motor control, of the kind: “sequence”-“primitives”-“muscle forces”.

However, anatomical studies of motor cortices have shown parallelity of these motor cortices. Unlike hierarchical connections from the SMA to the PM, and from the PM to the MI, these motor cortices are bidirectionally connected to each other, the majority of them moreover projecting directly to the spinal cord [53],[62]. These observations suggest that, despite strong evidence of functional hierarchy, these motor cortices do not possess clear anatomical hierarchical structure.

However, even without explicit hierarchical structure, the current neural network model study demonstrates that functional hierarchy of motor primitives and sequences of primitives can emerge through multiple timescales in neural activity. The idea of functional hierarchy that self-organizes through multiple timescales may as such contribute to providing an explanation for puzzling observations of functional hierarchy in the absence of an anatomical hierarchical structure.

### Multiple Scales in Space and Time: General Mechanisms for Hierarchy

At the conceptual level, it is intuitively understandable that forms of hierarchy can be realized through differing scales in space and time. In a photo image, for example, elemental information in a narrow space, such as the edges of an object and the color of pixels, is integrated into complex features of the image in a larger space. In speech sounds, syllable-level information on short time scale is integrated into word-level information over a longer time scale. It is not unrealistic to think that the mechanisms of multiple scales in space and time, which are responsible for generating these hierarchies, might also be at work in the neural systems of animals.

Information processing in the visual cortex, investigated extensively in the study of visual perception, is thought to occur on multiple spatial scales [63]–[65]. It is as such considered that functional hierarchy in visual information processing operates on the basis of the spatial structure of visual cortices, such as connections between local modules at a narrow spatial scale, and connections between brain regions at a wider spatial scale. This observation of functional hierarchy based on spatial hierarchy leads naturally to the idea of the local representation model.

On the other hand, there also exists a hypothesis claiming that hierarchical functional differentiation is caused by different timescales of neural activities, specifically, difference in the temporal integration window of neural activities. Based on the observation that speech perception requires multi-time resolution at the formant transition level (20–50 ms) and at the syllable level (200–300 ms), Poeppel [25] hypothesized that different temporal integration windows in neural activities correspond to a perceptual hierarchy between formant transition level and syllable level. In a neuroimaging study using auditory stimuli, Poeppel and his colleagues found that different brain regions responded in a way which corresponded to differences in the temporal properties of stimuli: one stimulus required precise temporal resolution and activated one particular brain region, while the other stimulus modulated the sound stimulus slowly and activated a different region [4].

It is also intuitively understandable that spatial scales of neural connectivity and timescales of neural activity work in concert with each other. Certain biological observations suggest that multiple scales in space and time in neural systems play an important role in giving rise to functional differentiation. For example, visual cortices of primates, considered to be organized according to a spatial hierarchy, also exhibit functional differentiation that is based on the timescales of neural activity. Many neurons in area V4, which is considered to process wavelength domains, fire in a sustained fashion (possibly to integrate longer time scale information); firing patterns in area MT/V5, on the other hand, which is considered to process visual motion perception, are phasic and brief in duration (possibly in order to achieve precise time resolution) [66].

There also exist studies emphasizing the relationship between spatial organization (neural connectivity) and the presence of multiple timescales in neural activity. For example, Honey et al. showed that, in simulations of a neural network that captured interregional connections of the macaque neocortex, neurons spontaneously synchronized at multiple timescales corresponding to local and global interactions in regions of the brain [24]. This study can be considered to have shown that multiple timescales can emerge in neural activity through constraints on connectivity. As mentioned earlier, Paine [27] showed that a particular constraint on connections encouraged the emergence in neural activity of functional hierarchy with multiple timescales. The model presented in this paper, which has a similar constraint on information flow, demonstrated that multiple timescales are an essential factor leading to the emergence of functional hierarchy. These facts strongly suggest that the spatial connections between neurons and the timescales of neural activity are strongly related to each other, and that both act as essential mechanisms leading to functional hierarchy in neural systems.

### Limitations of the Model and Questions for Future Research

The limitation of the current study results from simplicity of the system. The model network, for example, uses only 180 neurons abstracted to the level of a firing rate model. Input-output of the system consists of sensori-motor vectors with only 10 dimensions. Movement of the robot is constrained to 8 degree of freedom. Task behaviors in the current experimental environment were much more static than animal behavior in a real-life environment. Due to this simplicity of the system, discussing correspondences between the proposed model and an actual brain is possible only at a macro level of abstraction.

Despite these limitations, study of the proposed model marks important progress in advancing the synthetic approach. In the architecture proposed in the previous study by Paine [27], for example, it is difficult to increase the number of primitives that the model can learn, and it is likewise difficult to achieve a high dimensionality of sensori-motor control due to limitations on the number of parameters evolving in the learning process. In the current model, however, the model was able to learn more than twice the number of primitives learned by the model used in earlier studies [18],[27],[39]. In addition, the proposed network was successful in interacting robustly with a physical environment through the manipulation of a humanoid robot which had a higher dimensionality of sensori-motor control than that of the mobile robot used in the earlier study by Paine. An important issue for future research will be to investigate whether the proposed idea of functional hierarchy, which self-organizes through the operation of multiple timescales in neural activity, can be applied to a more biologically precise model using spiking neurons, or to a larger scale network carrying out more complex tasks.

## Method

### Robot Platform

The humanoid robot used in the current experiment was produced by Sony Corporation (video of robot experiment is available at: http://www.bdc.brain.riken.go.jp/~tani/mov/PLoS08.html). The robot is roughly 50 cm in height, with an arm span of about 30 cm. The robot was fixed to a stand, with tasks involving only movement of the head and arms of the robot. Each arm moves with 4 degrees of freedom (3 shoulders and 1 elbow) and the head motor moves with 2 degrees of freedom (vertical and horizontal).

The joints of the robot have a maximum rotation that ranges from 70 degrees to 110 degrees, depending on the type of joint. Rotation ranges were mapped to values ranging from 0.0 to 1.0. Encoder values of these arm joint sensors were received as the current proprioceptive sensory feedback and sent to the network. A vision system mounted on the robot's head automatically fixated a red mark on the object, regardless of the robot's actions. The direction of the robot's head, indicated by encoder values of two neck joints, expressed the object position in the visual field relative to the robot. This relative location of the object was treated as visual input to the system. When the robot received target joint angles, it automatically generated movements corresponding to these angles using a programmed proportional-integral-derivative (PID) controller. The sensory-motor state of the robot was sampled once every 150 msec. This sampling rate was the same as the numerical integration step interval of the CTRNN.

### Robot Performance Criteria

Each behavior of the robot was tested in every position. Performance was scored in terms of a success rate across all tasks. Criteria for failure or success were based on the reproduction of movement instructions from the teaching sequences, which included nearly the full range of joint angles. In tasks involving object manipulation (up-down, left-right and backward-forward), judgment of success depended on the robot not dropping the object during each movement. In tasks involving up-down behavior, success depended on whether the robot could repeat 3 times the action of lifting the object up to a height of 6 cm and bringing it down again. In left-right behavior, success depended on whether the robot was able to move the object left and right 3 times over a distance of more than 8 cm. In backward-forward behavior, success depended on whether the robot was able to move the object backward and forward 3 times over a distance of more than 6 cm. In the touch with single hand task, the robot had to reach the object with its right hand, within an error of no more than 1.0 cm. Finally, to succeed in the clapping hand behavior task, the robot had to bring its hands together 3 times. In all tasks, success also depended on whether the robot returned to its home position.

### Sparse Encoding of CTRNN Input-Output

**Figure 10:**
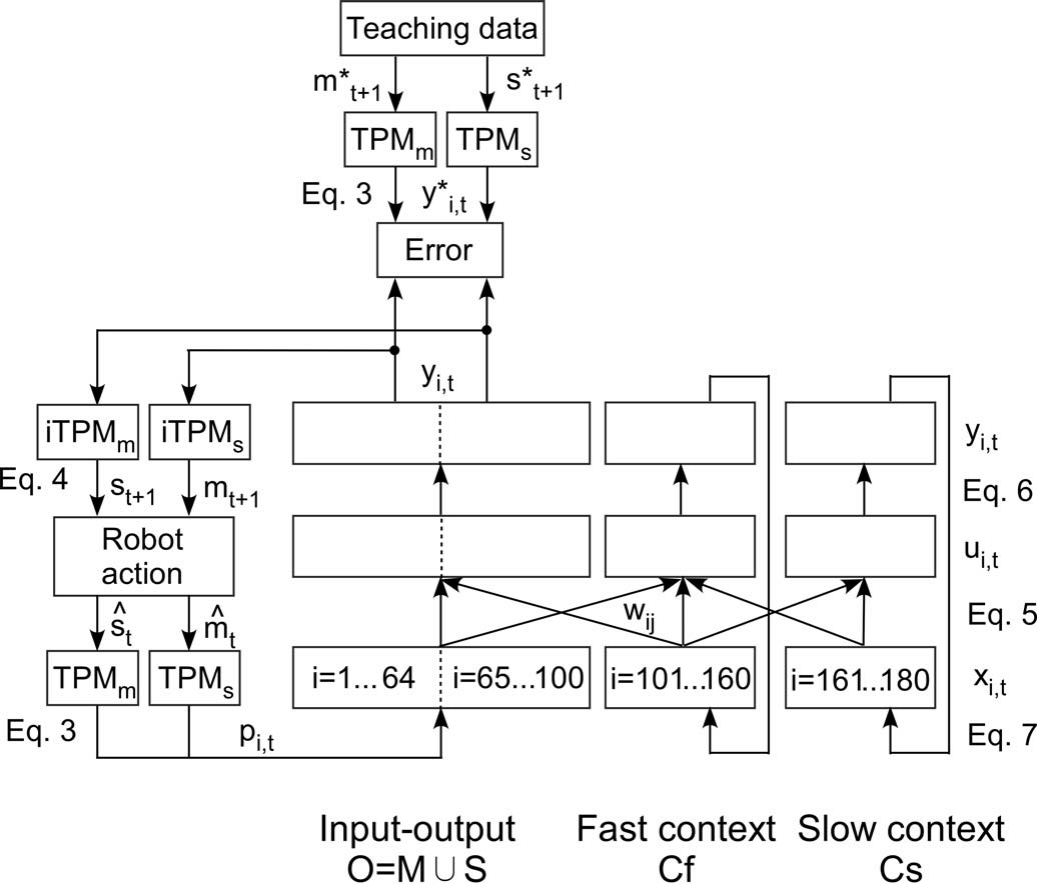
System details. The main part of the system is the CTRNN. The total number of CTRNN units was 180. The first 100 units (indices *i* = 1‥100) correspond to input-output units (*O*). Among input units, the first 64 units (indices *i* = 1‥64) correspond to proprioceptive inputs (*M*), whereas the last 36 units (indices *i* = 65‥100) correspond to vision inputs (*S*). The remaining 80 units (indices *i* = 101‥180) correspond to the context units. Among the context units, the first 60 units (indices *i* = 101‥160) correspond to the fast context units (*Cf*), and the last 20 units (indices *i* = 161‥180) correspond to the slow context units (*Cs*). Inputs to the system were the proprioception *mˆ* *_t_* and the vision sense *ŝ* *_t_*, which were transformed into sparsely encoded vectors using topology preserving maps (TPM, Equation 3), one map corresponding to proprioception (TPMm) and one map corresponding to vision (TPMs). A 100-dimensional vector, transformed by the TPM (*p_i,t_*) and previous activation levels of the context units *y_i,t_*_−1_, is set to the neural states *x_i,t_* (Equation 7). Membrane potential (*u_i,t_*) and activation (*y_i,t_*) of each unit are calculated using Equation 5 and Equation 6, respectively. Outputs of the CTRNN (*y_i,t_*, *i*∈*O*) are transformed into 10 dimensional vectors (*m_t_*_+1_ and *s_t_*_+1_) using inverse computation of the TPM (iTPM, Equation 4). These 10 dimensional vectors correspond to predictions of the proprioception *m_t_*_+1_ and the vision sense *s_t_*_+1_ for the next time step. This prediction of the proprioception *m_t_*_+1_ was sent to the robot in the form of target joint angles, which acted as motor commands for the robot in generating movements and interacting with the physical environment. Changes in the environment resulting from this interaction were sent back to the system in the form of sensory feedback. In training, output of the CTRNN (*y_i,t_*, *i*∈*O*) is compared with the desired output y*_i,t_ calculated from target sensori-motor states m*_t__+1_ and s*_t__+1_, using the same TPMs.

Inputs to the system were sparsely encoded in the form of a population coding using conventional topology preserving maps (TPM, [67]), one map corresponding to proprioception and one map corresponding to vision (Figure 10). The TPM is a type of a neural network that produces a discretized representation of the input space of training samples. The characteristic feature of the TPM is that it preserves topological properties of the input space. This sparse encoding of sensori-motor trajectories reduces the overlaps of sensori-motor sequences. The size of the TPMs were 64 (8×8) for proprioception and 36 (6×6) for vision sense, respectively. 10 dimensional proprioceptive and visual inputs were thus transformed into 100 dimensional sparsely encoded vectors.

In the current study, TPMs were trained in advance of CTRNN training using conventional unsupervised learning algorithm [67]. Samples for training of the TPMs included (1) all teaching sensori-motor sequences for the CTRNN, and (2) sensori-motor sequences for the set of all behavioral tasks performed at 2 cm in either direction beyond the standard position range (positions 0 and 6 in Figure 2B). This additional sample allowed the TPM to achieve a smooth representation of the input space and reduce loss of data incurred in the process of vector transformation. In the training of the TPM, data was sampled randomly, and training for both proprioception and vision TPMs was carried out over a total of 3×10^6^ epochs.

Reference vectors of the TPM are described as follows,
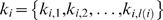
(2)where *l*(*i*) is dimension of the reference vector corresponding to the sample vectors of proprioception *m_t_* or vision *s_t_*. Thus *l*(*i*) is determined as follows: if *i*∈*M*, then *l*(*i*) = 8, and if *i*∈*S*, then *l*(*i*) = 2, where *M* and *S* are sets of indices corresponding to proprioception and vision.

The TPM transformation is described by following formula,
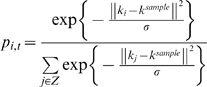
(3)where if *i*∈*M*, then *Z* = *M* and *k^sample^* = *m_t_*, if *i*∈*S*, then *Z* = *S* and *k^sample^* = *s_t_*. *σ* is a constant, indicating the shape of the distribution of *p_i,t_*, set at 0.01 in the current study. *p_i,t_* is a 100(64+36) dimensional vector transformed by the TPM which becomes the input to the CTRNN, the main component of the system.

The CTRNN generates predictions of next step sensory states based on the acquired forward dynamics described later. Outputs of the CTRNN were 100 dimensional vectors *y_i,t_*. The output of the CTRNN, assumed to correspond to an activation probability distribution over the TPM units, was again transformed into a 10 (8+2) dimensional vector using the same TPM:
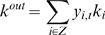
(4)where if *i*∈*M*, then *Z* = *M* and *k^out^* = *m_t_*_+1_, and if *i*∈*S*, then *Z* = *S* and *k^out^* = *s_t_*_+1_. These 10 dimensional vectors correspond to predictions of what values proprioception and vision will take at the next time step. *m_t_*_+1_ was sent to the robot as target joint angle.

### Action Generation Mode

The main part of the system studied in this paper is the CTRNN, which learns to generate temporal patterns of sensori-motor sequences (Figure 10). The number of CTRNN units *N* for this study was 180. The first 100 units (indices *i* = 1‥100) correspond to input-output units (*O*) which receive external input; their activation values *y_i,t_* correspond to output of the CTRNN. Among input units, the first 64 units (indices *i* = 1‥64) correspond to proprioceptive inputs (*M*), whereas the last 36 units (indices *i* = 65‥100) correspond to vision inputs (*S*). The time constant for the input-output units was set to 2.

The remaining 80 units (indices *i* = 101‥180) correspond to the context units (*C*). Among the context units, the first 60 units (indices *i* = 101‥160) correspond to the fast context units (*Cf*) with a small time constant value (*τ_i_* = 5), and last 20 units (indices *i* = 161‥180) correspond to the slow context units (*Cs*) with a large time constant value (*τ_i_* = 70). The number of input-output units is determined by the sizes of the TPMs. If the sizes of the TPMs are set to larger value, representations in the TPMs become smoother and data loss in the vector transformation decreases. For the current experiment, however, in order to reduce time spent on computation, sizes of the TPMs were selected such that they were the minimum value large enough to allow the TPMs to reproduce, in real time, sensori-motor sequences through the process of vector transformation. The number of context units was also selected to be the minimum value large enough to successfully allow the network to learn the task sequences. Larger numbers of context units was not found to increase performance of the model. The ratio between the number of fast and slow context units was set arbitrary and was not investigated in the current experiment.

Every unit of the CTRNN, with exceptions described later, is connected to every other unit, including itself. Values of connection weights are asymmetric, i.e. the weight value from the *j*th unit to the *i*th unit (*w_ij_*) is in general different from the weight value from the *i*th unit to the *j*th unit (*w_ji_*). Input units corresponding to proprioception and vision are not connected each other (if *i*∈*M* ∧ *j*∈*S*, or if *i*∈*S* ∧ *j*∈*M*, then *w_ij_* is fixed at 0). In addition, input units were not directly connected to slow context units (if *i*∈*O* ∧ *j*∈*Cs*, or if *i*∈*Cs* ∧ *j*∈*O*, then *w_ij_* is fixed at 0).

Neurons in the CTRNN are modeled according to a conventional firing rate model, in which the activity of each unit constitutes an average firing rate over groups of neurons. Continuous time characteristics of the model neurons are described by differential equation 1. Actual updating of *u_i,t_* values is computed according to Equation 5, which is the numerical approximation of Equation 1:
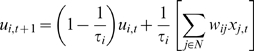
(5)The activation of the *i*th unit at time *t* (*y_i,t_*) is determined by the following formula:
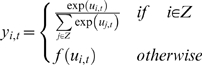
(6)where *Z* is *M* or *S*. Softmax activation is applied only to each group of output units (*M* and *S*), not to the context units. Activation values of the context units are calculated according to a conventional sigmoid function *f*(*x*)* = 1/1+e^−x^*. Application of softmax activation to the CTRNN makes it possible to maintain consistency with output of the TPM, which is calculated through use of the softmax function.

Activation values of output units are sent to the TPM and transformed into predictions of proprioception *m_t_*_+1_ and vision *s_t_*_+1_. Based on this prediction, the robot generates movement, as a result of which actual sensory feedback *mˆ* *_t_*_+1_ and *ŝ* *_t_*_+1_ are sent to the system and transformed into 100 dimensional vectors *p_i,t_*_+1_ using the TPMs described earlier. These 100 dimensional vectors are copied to *x_i,t_*_+1_ as external inputs to the CTRNN at the next time step. Activation values of the non-output units *y_i,t_*, one the other hand, are simply copied as recurrent inputs to the neural states of next time step, *x_i,t_*_+1_.
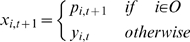
(7)


### Training Mode

A conventional back-propagation through time (BPTT) algorithm was used for training of the model network [45]. The objective of learning is to find optimal values of connective weights that minimize the value of *E*, defined as the learning error between the teaching sequences and output sequences. The error function *E* is determined using Kullback-Leibler divergence as follows,
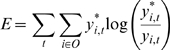
(8)where y*_i,t_ is the desired activation value of output units at time *t*. y*_i,t_ is calculated from the target sensory motor states m*_t__+1_, s*_t__+1_ using Equation 3.

Connective weights approach their optimal levels through a process in which values are updated in a direction opposite that of the gradient *∂E/∂w*.
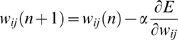
(9)where *α* is the learning rate constant, and *n* is an index representing the iteration step in the learning process. *∂E/∂w* is given by:
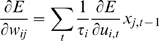
(10)and is recursively calculated from the following reccurence formula
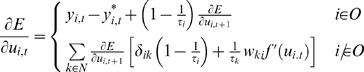
(11)where *f′*( ) is the derivative of the sigmoidal function and *δ_ik_* is Kronecker's delta (*δ_ik_* = 1 if *i* = *k* and otherwise 0).

A common problem in the BPTT algorithm arises from the difficulty of learning long temporal correlations in target sequences. This is due to error signals that are attenuated during the iterative process of back-propagation. In the proposed model, the large time constant value of the slow context units may have contributed to reduce attenuation of the propagated error signal.

Through iterative calculation of the BPTT algorithm, values of connective weights approach their optimal values, minimizing the error between teaching sequences and output sequences. Throughout the learning trials, the learning rate *α* is fixed at 5.0×10^−4^. Initial values of connective weights are set randomly to values ranging between −0.025 and 0.025.

In training mode, predicted values of *m_t_*_+1_ and *s_t_*_+1_ serve as virtual sensory feedback for the next time step *mˆ* *_t_*_+1_ and *ŝ* *_t_*_+1_ (mental simulation), rather than as sensory feedback from actual robot movements. In the process of this closed-loop training, error between generated sequences and teaching signals sometimes grows too large to estimate the gradient of the error landscape. To avoid this problem in learning, target sensori-motor state m*_t__+1_ and s*_t__+1_ are also incorporated into the predicted values of *m_t_*_+1_ and *s_t_*_+1_.
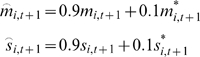
(12)


The portion of the target sensori-motor state incorporated into the predicted values of *m_t_*_+1_ and *s_t_*_+1_ was set by balancing with the learning rate. The setting of these parameters is not essential for model performance. As in the case of the generation mode, sensory feedback *mˆ* *_t_*_+1_ and *ŝ* *_t_*_+1_ are transformed into vectors *p_i,t_*_+1_ using the TPMs. The setting of the next time step *x_i,t_*_+1_ is same as in the case of the generation mode (Equation 7).

In order to reproduce different target behavior sequences, each target behavior is allocated a corresponding initial state in the slow context units as defined by the experimenter, based on the initial sensitivity characteristics of the CTRNN [44]. These initial state values were chosen in such a way as to maximize the distance between each behavior. On the other hand, both in training phase and action generation phase, initial states of the fast context units are always set to their neutral value, i.e. the internal state of each neuron is set to 0. Initial states of the input-output units are also set to the same value, corresponding to the home position for all task behavior.

### Additional Training of Novel Sequences

During additional training, only the connections of the slow context units were allowed to change. This corresponds to only allowing *w_ij_* to change in cases where *i*∈*Cs* ∧ *j*∈*Cf*, or *i*∈*Cf* ∧ *j*∈*Cs*, with other weights fixed at values generated through basic training. Initial states of slow context units were set to values that were different from those of the basic behavior patterns.

### PCA Analysis

For the PCA analysis of position generalization and of the additional training, different data sets were used. For the position generalization analysis, the data set included all basic behavior sequences for all object locations. To obtain PC conversion vectors, 60 dimensional vectors made up of fast context units and 20 dimensional vectors made up of slow context units were separately analyzed. For the PCA analysis of the additional training, in order to plot both the basic and novel behavior sequences in the same PC space, PC conversion vectors were calculated from the data set, which included all basic behaviors and two novel behavior sequences for all object locations. After calculation of the PC conversion vectors, basic behavior sequences and additional novel behavior sequences were separately transformed. Only the 60 dimensional vectors made up of the fast context units were used for the PCA analysis of the additional training. In all analyses, contributions of principal components 1, 2 and 3 were almost the same (about 15%). In all PCA analyses, principal components 1 and 3 were thus plotted based on their being the easiest to understand visually.
