# Correction: Emergence of Functional Hierarchy in a Multiple Timescale Neural Network Model: A Humanoid Robot Experiment

**DOI:** 10.1371/annotation/c580e39c-00bc-43a2-9b15-af71350f9d43

**Published:** 2010-10-06

**Authors:** Yuichi Yamashita, Jun Tani

In the Method section there is an error in Equation 11. Please view the correct equation here: 


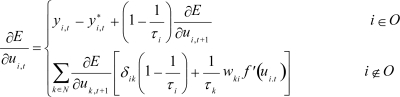


In the lower part of the Equation 11, after summation over k, dE/du_i,t+1 (error) should be dE/du_k,t+1 (correct).

